# Diffusion tensor imaging discriminates focal cortical dysplasia from normal brain parenchyma and differentiates between focal cortical dysplasia types

**DOI:** 10.1186/s13244-023-01368-y

**Published:** 2023-02-24

**Authors:** Antonio Giulio Gennari, Dorottya Cserpan, Ilona Stefanos-Yakoub, Raimund Kottke, Ruth O’Gorman Tuura, Georgia Ramantani

**Affiliations:** 1 Department of Neuropediatrics, University Children’s Hospital Zurich, Steinwiesstrasse 75, 8032 Zurich, Switzerland; 2 MR-Research Centre, University Children’s Hospital Zurich, Zurich, Switzerland; 3 Department of Diagnostic Imaging, University Children’s Hospital Zurich, Zurich, Switzerland; 4 University of Zurich, Zurich, Switzerland; 5 Children’s Research Centre, University Children’s Hospital Zurich, Zurich, Switzerland

**Keywords:** Paediatric focal epilepsy, Focal cortical dysplasia, DTI, MRI, Malformation of cortical development

## Abstract

**Objectives:**

Although diffusion tensor imaging (DTI) may facilitate the identification of cytoarchitectural changes associated with focal cortical dysplasia (FCD), the predominant aetiology of paediatric structural epilepsy, its potential has thus far remained unexplored in this population. Here, we investigated whether DTI indices can differentiate FCD from contralateral brain parenchyma (CBP) and whether clinical features affect these indices.

**Methods:**

In this single-centre, retrospective study, we considered children and adolescents with FCD-associated epilepsy who underwent brain magnetic resonance (MRI), including DTI. Fractional anisotropy (FA), mean diffusivity (MD), axial diffusivity, and radial diffusivity, were calculated in both FCD and CBP. The DTI indices best discriminating between FCD and CBP were subsequently used to assess the link between DTI and selected clinical and lesion-related parameters.

**Results:**

We enrolled 32 patients (20 male; median age at MRI 4 years), including 15 with histologically confirmed FCD. FA values were lower (*p* = 0.03), whereas MD values were higher in FCD than in CBP (*p* = 0.04). The difference in FA values between FCD and CBP was more pronounced for a positive vs. negative history of status epilepticus (*p* = 0.004). Among histologically confirmed cases, the difference in FA values between FCD and CBP was more pronounced for type IIb versus type I FCD (*p* = 0.03).

**Conclusions:**

FA and MD discriminate between FCD and CBP, while FA differentiates between FCD types. Status epilepticus increases differences in FA, potentially reflecting changes induced in the brain. Our findings support the potential of DTI to serve as a non-invasive biomarker to characterise FCD in the paediatric population.

**Supplementary Information:**

The online version contains supplementary material available at 10.1186/s13244-023-01368-y.

## Key points

1. DTI derived indices may be a useful tool for FCD characterisation.
2. FA and MD discriminated between FCD and contralateral brain parenchyma (CBP).
3. Status epilepticus increased the FA difference values between FCD and CBP.
4. Type IIb showed higher FA difference values than type I FCD.

**Supplementary Information:**

The online version contains supplementary material available at 10.1186/s13244-023-01368-y.

## Background

Epilepsy is the most common diagnosis in children hospitalised due to neurological diseases [1]. Roughly 20% of children with epilepsy will experience pharmacoresistance [2], defined as the failure of adequate trials of (at least) two tolerated anti-seizure drugs (ASD) to achieve sustained seizure freedom [3]. The combined impact of the underlying pathology, uncontrolled seizures, and ineffective ASD on the developing brain [4] can result in significant comorbidities, such as developmental and behavioural issues, psychiatric problems, and poor quality of life. The presence of a brain lesion is the most critical predictor of pharmacoresistance [2], which usually manifests at an early stage [5]. For children with pharmacoresistant focal structural epilepsy, surgery is the only treatment that offers the potential of cure, as recently shown in a randomised controlled trial [6]. Focal cortical dysplasias (FCDs) are congenital brain abnormalities that represent the most common cause of paediatric pharmacoresistant focal epilepsy amenable to surgery [7], particularly in early life [8, 9]. However, both the referral for presurgical evaluation and postsurgical seizure freedom [10] depend heavily on the accurate identification, delineation, and differentiation of FCD in magnetic resonance imaging (MRI) [11].

Despite the improved lesion detection rates due to recent advances in neuroimaging, FCD remains the most common epilepsy substrate escaping detection by MRI [12], with MRI sensitivity and specificity for FCD detection limited to 62% and 77%, respectively [13]. FCDs are divided into three types according to the presence of different histologic hallmarks [14] that determine their MRI-detectability [13]. Preliminary studies have suggested that diffusion tensor imaging (DTI) can depict differences between the brains of adults with malformations of cortical development and those of controls, invisible on conventional MRI sequences [15]. DTI reflects the diffusion of extracellular water molecules, thus rendering DTI metrics sensitive to abnormalities in tissue cytoarchitecture and microstructure. However, to date, only a few studies have employed the scalar indices derived from DTI, namely fractional anisotropy (FA), mean (MD), radial (RD), and axial diffusivity (AD), to study FCD, yielding contradictory results [16–20]. Moreover, most of these studies have been conducted in predominantly adult cohorts that included small subsets of 2 to 24 children and (mainly) adolescents, thus calling into question the applicability of this approach in purely paediatric cohorts and the validity of these results across the age spectrum. Indeed, in addition to changes in myelination, many other processes differentiate children’s brains from adult brains, such as tubulinogenesis, axonogenesis and synaptogenesis in the postnatal period [21], regression of grey matter volume in late childhood [22], as well as pubertal rewiring of brain circuits and dendritic pruning in adolescence [23]. Furthermore, to the best of our knowledge, no previous studies have compared DTI indices in paediatric focal structural epilepsy with lesion characteristics and clinical features, particularly those reflecting epilepsy severity.

In the present study, we aimed to determine (1) whether DTI indices facilitate the characterization of FCD in paediatric focal structural epilepsy and (2) whether these DTI indices are impacted by lesion characteristics and by epilepsy severity. To address these hypotheses, we compared the DTI indices between the FCD and the contralateral brain parenchyma (CBP) in one of the largest paediatric cohorts. Also, we investigated the interrelation of the relevant DTI indices with lesion characteristics and clinical features reflecting the state of the disease.

## Methods

### Patient selection

**Fig. 1:**
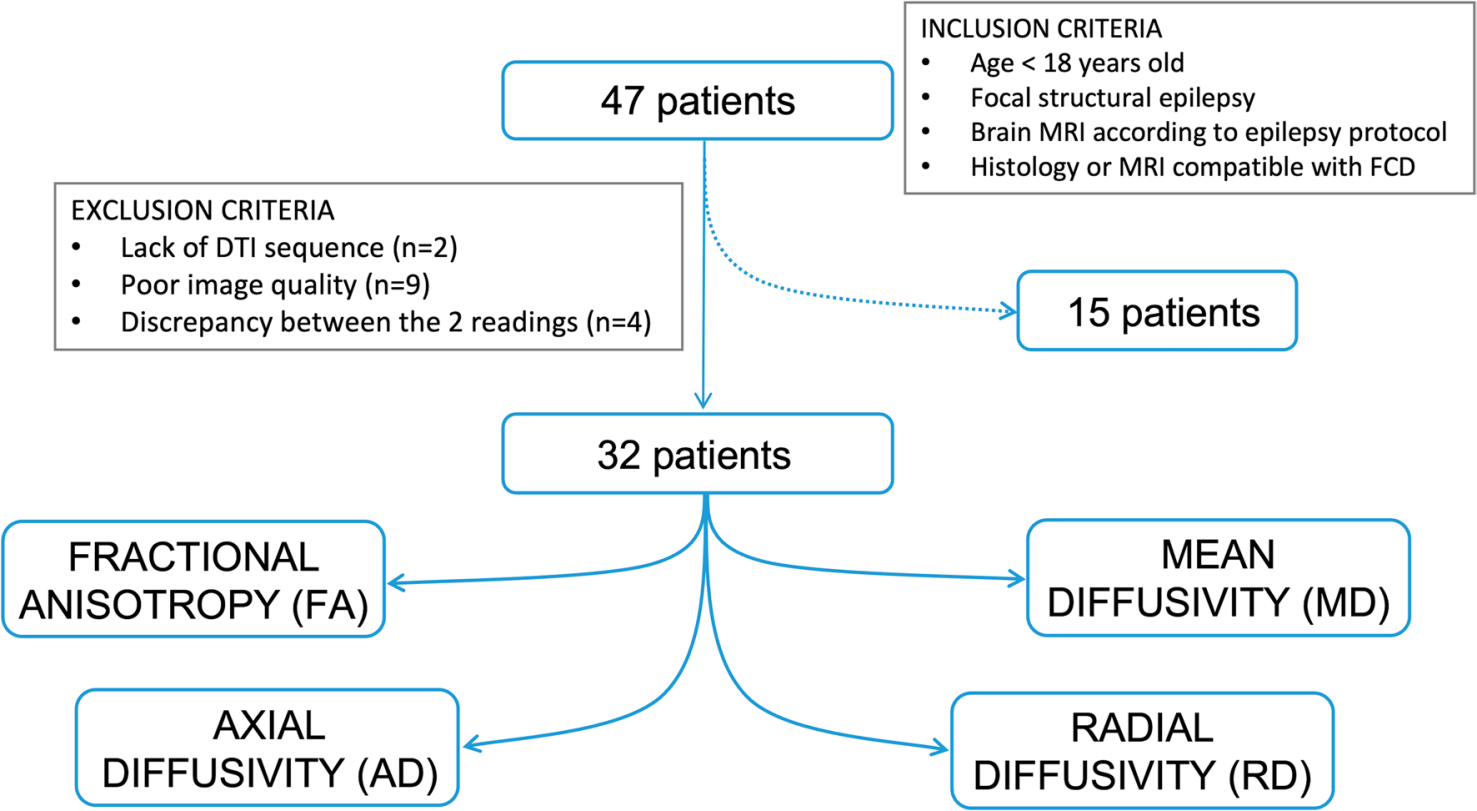
Flow diagram of our patient selection

In this single-centre retrospective study, we selected consecutive children and adolescents who underwent MRI at the University Children’s Hospital Zurich, according to a dedicated epilepsy protocol, between the 1st of January 2007 and the 1st of November 2021. Inclusion criteria for our study were: (1) diagnosis of focal structural epilepsy based on electroclinical correlations and the presence of an MRI-detectable lesion, (2) MRI reports compatible with FCD according to previously defined and widely adopted criteria [24], and (3) age < 18 years at the time of the scan. An experienced radiologist with several years of clinical and research experience in neuroradiology (A.G.G.) with an experienced neuropaediatrician and epileptologist (G.R.), blinded to the original MRI reports, performed a second reading session, in consensus, thoroughly reviewing the MRI exams of patients who met the inclusion criteria. The results of this additional reading session were compared to the original MRI reports. Exclusion criteria were: (1) poor image quality in patients who underwent a single MRI scan, (2) discrepancies between the two reading sessions, and (3) lack of a DTI sequence (Fig. 1). Our final patient cohort consisted of 32 children and adolescents (20 male) aged 1 to 16 years (median: 4 years, IQR: 2 to 11) at the time of the MRI scan relevant for our analysis. The collection of patient data and the scientific analysis were approved by and performed according to the guidelines and regulations of the local ethics committee (Kantonale Ethikkommission Zürich, KEK-ZH PB-2019-01854). All patients and their caretakers gave written informed general consent to re-use their clinical and MRI data for research.

### Imaging protocol

All MRI scans were acquired on a 3T scanner (Signa EXCITE HD.xt^@^, upgraded to Discovery MR 750^@^, GE Medical Systems, WI, USA) using an 8-channel head coil. All patients underwent a 3D-fast spoiled gradient echo (FSPGR) T1-weighted image (T1WI) sequence and a DTI sequence. Sagittal, coronal, and axial T2-weighted image (T2WI), as well as coronal and axial fluid-attenuated inversion recovery (FLAIR), and/or 3D-FLAIR, were also included in our institutional protocol. Image parameters are detailed in Additional file 1: Table S1.

DTI sequences were acquired according to two different DTI protocols, depending on the date of acquisition:

1. Single-shell 21 direction protocol (4 patients; exams acquired on the HD.xt scanner before November 2012).
2. Single-shell 35 direction protocol (28 patients; exams acquired on the MR750 scanner after November 2012).

### FCD segmentation

FCD segmentation was performed on the most recent MRI scan depicting the epileptogenic lesion in each patient, which corresponded to the last presurgical scan in patients who had undergone epilepsy surgery. Segmentation was performed on the next most recent scan in cases where multiple MRI scans were acquired and artefacts flawed the most recent scan. Both FCD segmentation and DTI analysis were performed on the same scan for each patient.

3D-FSPGR T1WI and 3D/2D FLAIR/T2WI were loaded into Slicer^@^ (https://www.slicer.org), synchronised and overlaid. Before starting the segmentation, hemispheric and lobar involvement were annotated. Lobar involvement was classified as: “frontal”, “temporal”, “posterior”, including parietal and occipital lesions, and “multilobar”. “Multilobar” lesions included more than one lobe irrespective of the lobes involved.

A 3D binary mask was generated by drawing a freehand, polygonal region of interest (ROI) outlining the FCD intensity changes seen on T1WI and FLAIR/T2WI. In accordance with clinical neurosurgical practice [25] and previous studies [16, 18, 19], both grey and white matter were included in the ROI. Minor segmenting errors were eliminated using a dilation-erosion algorithm before smoothing the ROI shape. The position and profiles of the binary mask were carefully compared to those of the brain gyri and modified as needed. Finally, the FCD volume was calculated from the volume of the segmented area.

### MRI post-processing

**Fig. 2:**
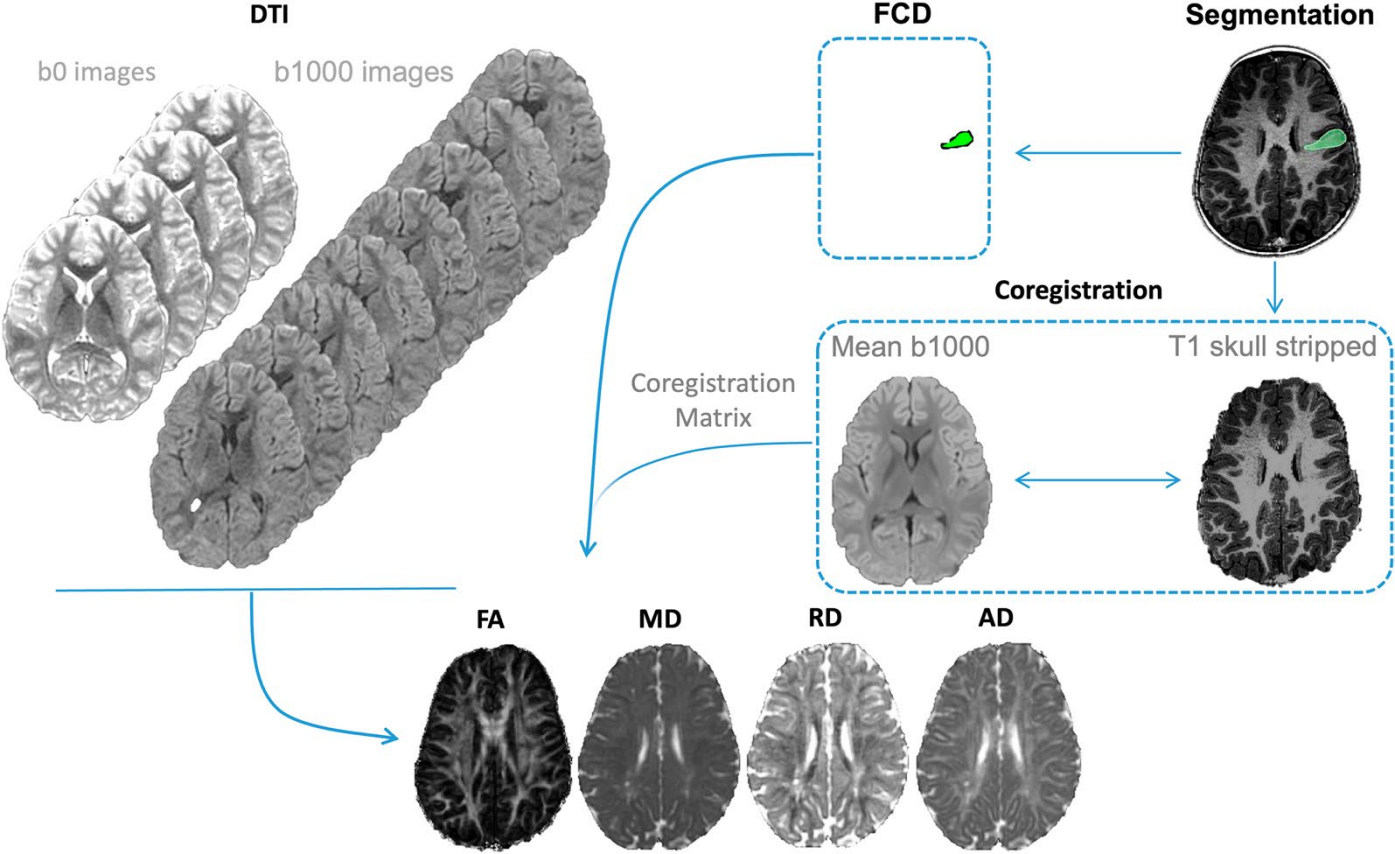
Image processing workflow. Anatomical images were used to manually segment FCD, leading to binary label generation. The FA, MD, RD, and AD maps were derived from DTI raw data; subsequently, b0 were separated from b1000 images. A mean b1000 volume was created and co-registered to the anatomical images. The coregistration matrix derived from the previous step allowed the alignment of the anatomical images, the binary label, and the above maps. The average FA, MD, AD, and RD values within the binary label were calculated, and then the label was flipped to the contralateral side to evaluate the CBP. *FCD:* Focal cortical dysplasia, *CBP*: Contralateral brain parenchyma, *FA*: Fractional anisotropy, *MD*: Mean diffusivity, *AD*: Axial diffusivity, *RD*: Radial diffusivity

FSL (www. fmrib.ox.ac.uk/fsl) was used to pre-process both 3D-FSPGR T1WI and DTI images. Post-processing steps are detailed in Additional file 1 (Fig. 2).

### Statistical analysis

All statistical analyses were performed using R software version 1.4.1106 (https://www.r-project.org). Continuous variables were reported as mean ± standard deviation (SD) or median (interquartile range, IQR), as appropriate, while categorical variables were presented as absolute numbers and percentages. D’Agostino-Pearson test was used to assess data skewness. Parametric, nonparametric, Chi-Square, and Fisher’s exact test were used to compare the distribution of normally distributed, non-normally distributed, and categorical variables, respectively. Results were presented as effect and Confidence interval (CI).

For each DTI map, the within ROI average value of the FCD was calculated and compared to that of the homologous CBP as done by previous studies on this topic [18–20]. The Youden index method was applied to the scalar index showing the most significant difference between FCD and CBP, aiming to identify the cut-off value with the combined highest sensitivity and specificity in discriminating between the two. The DTI indices best discriminating between FCD and CBP were then entered in the analysis to assess the link between these DTI changes and selected clinical (sex, epilepsy duration, age at MRI, seizure frequency, history of status epilepticus, and total ASD trials) and lesion related parameters (lesion lateralisation, lesion localisation, and lesion volume), using the difference in index between the two regions [18]. FCD subtype profiling analysis was limited to the patients with histologically confirmed FCD (14 patients subdivided as follows: 7 type I, 4 type IIa, and 3 type IIb), excluding one patient with type IIIb FCD. The analysis was restricted to the DTI scalar index best discerning between the lesion and CBP, using the same approach as described above. Statistical significance was set at *p* < 0.05. Holm correction was used in the case of multiple comparisons.

## Results

### Clinical features

The age at epilepsy onset in our cohort of 32 children and adolescents was 0 to 13 years (median: 1 year, IQR: 0 to 4), while the epilepsy duration was 0 to 16 years (median: 2 years, IQR: 0 to 4). At the time of the MRI scan, eight (25%) patients had been seizure-free for one year or longer, whereas 11 (34%) had daily seizures. Seven (22%) patients had a history of status epilepticus (SE), with a median latency of 8 months (IQR: 0 to 52 months) between the most recent episode of SE and the MRI considered for analysis in our study. The latency between SE and MRI was one week in two cases but no MRI was performed within 24 h from SE. It should be noted that two of eight seizure-free patients and three of 11 patients with daily seizures had a positive history of SE. Fifteen of 32 (47%) patients eventually underwent resective epilepsy surgery. Clinical features did not differ statistically between the subgroups of surgical and non-surgical patients, except for the total number of ASD trials, since patients undergoing epilepsy surgery had failed 1–8 (median: 5) ASDs at the MRI scan, compared to 0–1 ASDs in conservatively managed patients (median: 1, *p* = 0.003). Table 1 and Additional file 1: Table S2 present the clinical features of our patient cohort.

**Table 1:** Clinical features of our cohort

Clinical features	Non-surgical patients (*N* = 17)	Surgical patients (*N* = 15)	*p*-value	All patients (*N* = 32)
Male, *n* (%)	12 (71%)	8 (53%)	1	20 (63%)
Age at epilepsy onset in *y*, median (IQR)	2 (1 to 7)	0 (0 to 2)	0.64	1 (0 to 4)
Epilepsy duration in *y*, median (IQR)	1 (0 to 4)	2 (0 to 3)	1	2 (0 to 4)
Age at MRI in *y*, median (IQR)	8 (3 to 11)	3 (2 to 6)	0.56	4 (2 to 11)
Seizure frequency per month, median (IQR)	0.3 (0 to 90)	16 (4 to 300)	0.28	9 (0.1 to 158)
Positive history of status epilepticus, *n* (%)	5 (29%)	2 (13%)	1	7 (22%)
Total ASD trials, median (IQR)	1 (0 to 1)	5 (3 to 6)	0.003*	2 (1 to 5)

*n*: number, *y*: years, *IQR*: Interquartile range, *ASD*: Anti-seizure drugs, *MRI*: Magnetic resonance imaging

*p < 0.05 statistical significance

### Lesion characteristics

Lesion lateralisation was left in 13 (41%) patients. Lesion extent was unilobar in 88% and multilobar in 12% of patients. Among unilobar lesions, 16 (57%) were frontal, 7 (25%) temporal, and 5 (18%) posterior. The median volume was 11,309 mm^3^ for temporal, 7144 mm^3^ for frontal, 5986 mm^3^ for posterior lesions (5494 mm^3^ for parietal and 19911 mm^3^ for occipital lesions, respectively), and 15553 mm^3^ for multilobar lesions. Additional file 1: Table S3 illustrates the lesion characteristics of our cohort.

Histopathology verified FCD type I in 7 (47%), type IIa in 4 (27%), type IIb in 3 (20%) patients, and type IIIb (FCD associated with ganglioglioma) in one patient.

### DTI indices

**Fig. 3:**
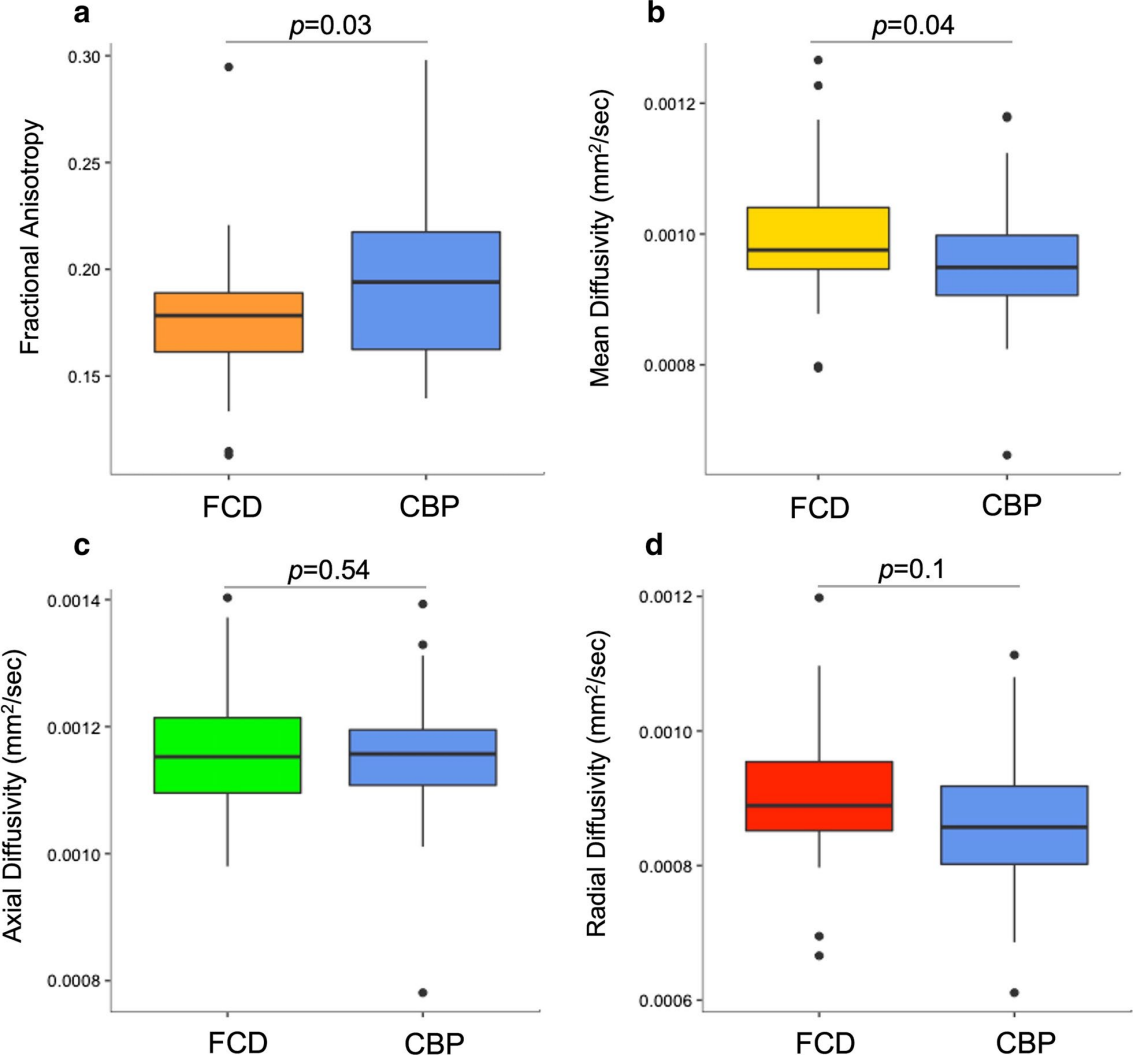
Boxplots presenting the difference in the DTI scalar indices between FCD and CBP. **a** FA values in FCD were lower than in CBP (median 0.18, IQR: 0.16 to 0.19; CBP: median 0.19, IQR: 0.16 to 0.22), while (**b**) MD values in FCD were higher than in CBP (FCD: median 0.00098, IQR: 0.00095 to 0.00104; CBP: median 0.00095, IQR: 0.00091 to 0.00100). No statistical differences in (**c**) AD and (**d**) RD were found between FCD and CBP (AD FCD: median 0.0012, IQR: 0.0011 to 0.0012; CBP: median 0.0012, IQR: 0.0011 to 0.0012; RD FCD: median 0.00089, IQR: 0.00085 to 0.00095; CBP: median 0.00086, IQR: 0.00080 to 0.00092). *FCD*: Focal cortical dysplasia, *CBP*: Contralateral brain parenchyma, *FA*: Fractional anisotropy, *MD*: Mean diffusivity, *AD*: Axial diffusivity, *RD*: Radial diffusivity, *DTI*: Diffusion tensor imaging, *IQR*: Interquartile range, *mm*: millimetres, *sec*: seconds

FA values were lower in FCD than in CBP (*p* = 0.03), whereas MD values were higher in FCD than in CBP (*p* = 0.04) (Fig. 3A, B). In contrast, AD and RD values did not differ between FCD and CBP (AD: *p* = 0.54; RD: *p* = 0.1, Fig. 3C, D). The optimal FA threshold for differentiating FCD from CBP, derived by the Youden index approach, was 0.19, with a sensitivity, specificity, and an area under the receiver operating characteristic curve of 81%, 56%, and 0.63, respectively. None of the DTI indices calculated in FCD or CBP correlated with lesion volume or patient age at MRI (Additional file 1: Fig. S1). Also, FA values in FCD and CBP did not differ between children older and younger than 3 years of age (*p* = 0.84 and *p* = 0.44, respectively). Similarly, MD values of FCD did not differ in the same age categories (*p* = 0.54), while those calculated in the CBP did (*p* = 0.02), as expected by previous literature [26].

**Fig. 4:**
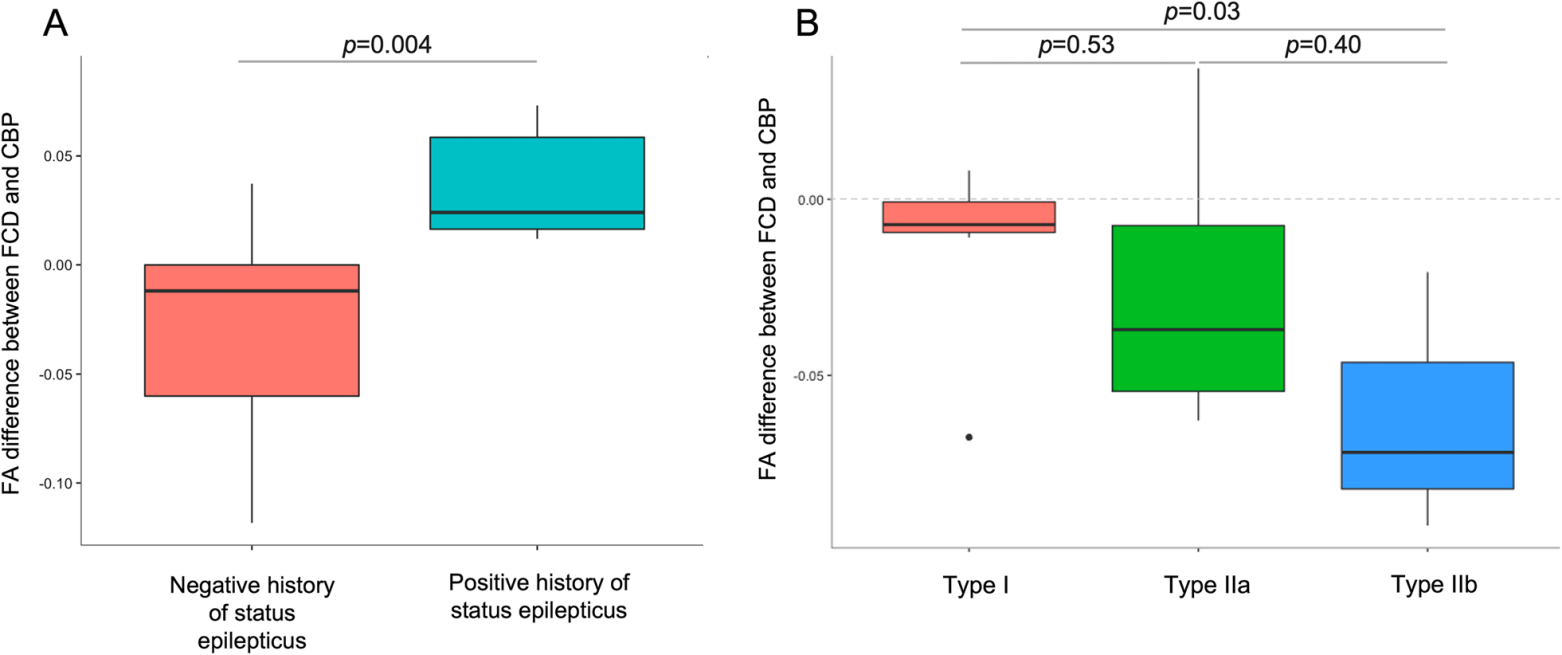
Boxplots presenting the difference in FA between FCD and CBP according to history of status epilepticus and FCD types. **A** In our cohort of 32 patients, the FA difference values between FCD and CBP were higher in patients who experienced at least one episode of status epilepticus in their life (median: 0.020, IQR: 0.013 to 0.059) compared to patients who did not (median: − 0.012, IQR: − 0.064 to 0.00086). **B** Among 14/15 patients with histologically proven FCD, the FA difference values between FCD and CBP were higher in FCD type I compared to type IIb (type I: median − 0.007, IQR: − 0.0008 to − 0.009; type IIb: median − 0.07, IQR: − 0.05 to − 0.08). Similarly, FA difference values were higher in FCD type I than in type IIa, but this difference did not reach statistical significance (type IIa: median: − 0.037, IQR: − 0.007 to − 0.05). *FCD:* Focal cortical dysplasia, *CBP:* Contralateral brain parenchyma, *FA:* Fractional anisotropy, *IQR:* Interquartile range

Among all 32 patients, those with a positive history of SE had significantly higher FA difference values between FCD and CBP than those with a negative history of SE (*p* = 0.004, Fig. 4A). Sex, epilepsy duration, age at MRI, seizure frequency, total ASD trials, and lesion lateralisation, localisation, and volume did not significantly impact FA difference values. Table 2 presents the results comparing FA difference values to clinical parameters.

**Table 2:** Correlation analysis of FA difference values with respect to clinical features and lesion characteristics

Clinical features and lesion characteristics	FA difference: FCD versus CBP	Correlation coefficient	*p*-value
Sex, median (IQR)^#^			1
Male	0.008 (− 0.006 to 0.017)		
Female	0.049 (− 0.001 to 0.069)		
Epilepsy duration in y^§^		− 0.19 (− 0.57 to 0.18)	1
Age at MRI in y^§^		− 0.15 (− 0.51 to 0.21)	1
Seizure frequency per month^§^		0.09 (− 0.22 to 0.43)	1
History of status epilepticus, median (IQR)^#^			0.004*
Positive	0.012 (− 0.042 to 0.021)		
Negative	0.011 (− 0.00026 to 0.063)		
Total ASD trials^§^		− 0.12 (− 0.47 to 0.24)	1
Lesion lateralisation, mean ± SD^+^			1
Right	0.020 ± 0.051		
Left	0.018 ± 0.039		
Lobar localisation, median (IQR)^#^			0.79
Temporal	0.013 (0.003 to 0.046)		
Extratemporal	0.003 (− 0.016 to 0.013)		
Lesion volume in mm^3§^		− 0.11 (− 0.44 to 0.29)	1

*FA*: Fractional anisotropy, *FCD*: Focal cortical dysplasia, *CBP*: Contralateral brain parenchyma, *IQR*: Interquartile range, *y*: years, *ASD*: Anti-seizure drugs

^#^Mann–Whitney U test

^+^Welch t test

^§^Spearman correlation coefficient

*p < 0.05 statistical significance

Among 14 (93%) of the surgically confirmed FCD cases, FA difference values were considerably higher for FCD type IIb than for FCD type I (*p* = 0.03), while FA difference values were similar for FCD type I and type IIa (*p* = 0.53) and for FCD type IIa and type IIb (*p* = 0.40) (Fig. 4B).

## Discussion

To the best of our knowledge, this is the first large paediatric cohort study to demonstrate that FA and MD can facilitate the discrimination between FCD and CBP, as well as between specific FCD subtypes, in paediatric focal epilepsy. Our results suggest that DTI may provide a powerful tool for FCD characterisation across the age spectrum, including the particularly vulnerable subgroup of very young children with focal structural epilepsy.

Although diffusion MRI has the potential to image microstructural changes within FCD and thus facilitate their differentiation from normal brain parenchyma, this promising technique has received little attention in previous FCD detection and characterisation studies that focused mainly on structural MRI [27]. Compared to anatomical images, diffusion MRI techniques offer a lower spatial resolution that limits their utility for lesion detection and delineation, which usually represents the primary outcome of radiological studies. However, the quantitative MRI assessment provided by DTI indices may help confirm subtle findings detected on anatomical images, such as those characterising FCD, thus resolving the ambiguity introduced by the inter-rater variability inherent in visual assessment. The present study reports several new and innovative findings in paediatric focal epilepsy, underscoring the potential utility of diffusion imaging techniques in this population.

### FA and MD can discriminate FCD from the normal brain parenchyma

The reduction in the FA values and the increase in the MD values in the FCD region compared to CBP, as detected in our study, is in line with previous work [15, 28, 29]. Of note, FA provided the most robust discrimination between the various DTI metrics. This difference in DTI indices between FCD and CBP can be attributed mainly to the higher diffusivity of water molecules in FCD due to changes in white matter microstructure, including myelin loss, abnormal myelin sheet formation, neuronal death, diminished arborisation of dendrites and reactive gliosis induced by recurrent seizures [19, 30]. The impact of FCD-related myelin changes on DTI indices is further supported by anatomical MRI studies in different histopathological substrates, which showed that over 80% of the overall MRI signal reflects myelin density variation, even in different cortical layers [31]. Finally, although it would be tempting to attribute the DTI changes established in our study to the specific cytological alterations characterising the different FCD types, it should be noted that the large slice thickness used in the majority of DTI clinical protocols (2.5 mm) corresponds to the average cortical thickness [32], thus limiting the possibility to image subtle cortical changes.

Interestingly, our observations contrast with a previous study in paediatric FCD-associated epilepsy [18] that found no FCD-specific signal changes in FA and MD indices. However, in this previous cohort, roughly 70% of patients had histological findings and in one-third of them histopathology ruled out the presence of FCD [18]. Moreover, the discrepancy between the two studies may also be attributed to the differences in their methodology, since the diffusion values in this past study were sampled at steps of 0.5 mm down to 6 mm from the pial surface [18], with more extensive FCD-specific signal changes at sampling depths below 2.5 mm. In contrast, we chose to estimate the mean diffusion value within a predefined ROI. Although interesting, the intricate approach used in this past study [18] is time-consuming, requiring additional pre-processing steps, and may offer only a limited advantage over our more straightforward approach, considering the limitations posed to analysis precision by the 2 mm slice thickness of diffusion images.

### FA values vary according to the history of status epilepticus

In our study, patients who experienced at least one episode of SE showed higher differences in FA between FCD and CBP. Therefore, these findings may reflect changes induced in the brain by such a disruptive event and are consistent with reports from animal studies that have previously demonstrated changes induced by a single episode of SE in the rat hippocampus [33, 34]. Interestingly, the FA of the dentate gyrus in animals with a history of SE has been considerably higher than in both healthy animals [33] and animals with a history of traumatic brain injury [34]. Histological analyses attributed these changes to an increase in astrocytes in the affected areas [33] without a concomitant increased vascularisation, suggesting that the increase in FA values is unlikely to be related to spurious signals from newly emerging vessels [35]. These results corroborate previous studies supporting that FA increases at the presence of astrogliosis and glial fibrillary acid protein deposition [36]. While no direct comparisons between animal findings and our results can be drawn, our results strongly motivate further investigation on this topic in a larger patient population, comparing diffusion imaging to histological analysis.

### FA can distinguish between FCD subtypes

Although based on a limited number of patients, our findings underline the potential of FA difference values in facilitating the distinction between FCD types, particularly between types I and IIb, which represent the two ends of the FCD severity spectrum. This finding is crucial since neurite orientation dispersion and density imaging (NODDI) and spherical mean technique (SMT) have been so far the only diffusion-based techniques to differentiate between FCD subtypes in paediatric focal epilepsy [18]. Here, it is essential to note that DTI reflects myelin changes, whereas NODDI and SMT additionally reflect intra- and extracellular neuropathological processes. Therefore, DTI may facilitate distinguishing type I and type II FCD based on their different effects on myelin [37], and NODDI and SMT may provide complementary information differentiating type IIa and IIb based on their specific cytological alterations [18]. In parallel with a visual MRI assessment, quantitative DTI indices may therefore provide a valuable tool for discriminating between FCD types, thus facilitating patient management, counselling and prognostication in all patients with FCD-associated focal epilepsy, irrespective of their candidacy for epilepsy surgery [38]. Moreover, in patients eventually undergoing epilepsy surgery, DTI indices could be combined with genetic markers to refine the characterisation of FCD types by histopathology [39, 40]. It should be noted that genetic studies in FCD rely on the availability of resected brain tissue that has considerably decreased over time, in line with the increased implementation of minimally invasive surgical techniques, such as laser interstitial thermal therapy, thus impeding histological evaluation in these patients [41]. Therefore, in the future, diffusion-weighted sequences and other MRI methods sensitive to cytological alterations may become increasingly important for the definition of FCD types. However, one potential confound to consider in the assessment of FA difference values within FCDs is that of astrogliosis, since histopathological assessments described various grades of astrogliosis within FCDs [42], and the severity appears to be linked to epilepsy activity rather than to FCD type [42]. Since astrogliosis may affect FA difference values, further studies exploring the effects of epileptic activity on imaging and histopathological findings in FCD are needed before implementing this promising tool in the diagnostic workup of these patients.

Our results, including those related to SE-specific changes in DTI metrics, derive from a paediatric cohort strongly focusing on the first years of life, thus reflecting the characteristics of this particularly vulnerable age group. Roughly one-half of patients who had experienced SE in our cohort were aged three years or younger at the MRI scan. Moreover, one-half of all patients were aged three years or younger at enrolment and one-half of surgically treated patients were diagnosed with FCD type I. Type I FCD manifests as early-onset epilepsy, often taking a refractory course [5] that may account for the very young age at presentation and comprehensive presurgical evaluation, including imaging, in our cohort. Although these characteristics underline the representativity of our cohort for the paediatric population undergoing presurgical assessment and, eventually, epilepsy surgery [8, 9, 43–45], more extensive multicentric studies are required to investigate the applicability of our findings across the paediatric age spectrum and refine the accuracy of our observations.

### Limitations

Our study has several limitations. Firstly, it is a retrospective, single-centre study, with all the inherent limitations of this study design. However, it should be noted that our findings derive from a large homogeneous cohort extending across the paediatric age spectrum with one-half of patients aged three years or younger, thus supporting the representativity of our cohort for the paediatric population with FCD-associated epilepsy undergoing presurgical evaluation and, eventually, epilepsy surgery. Secondly, we considered data acquired using two different DTI protocols, before and after a major upgrade of the scanner. However, a previous study has reported similar FA and MD values in healthy volunteers who underwent various DTI protocols with varying numbers of gradient directions [46]. Moreover, a simulation study showed that the variability in FA values asymptotically decreases as the number of gradient encoding directions increase; a gradient scheme with 20 encoding directions were deemed the point of minimal effect, in which FA difference with increasing gradient directions become negligible [47]. Thirdly, the inclusion of children < 3 years old in our analysis might have biased our results. However, this age category has already been included in the work of other authors, although to a lesser extent [18], and by using a region of healthy brain parenchyma as control region, we endeavoured to control for age-related changes in diffusion indices. Additionally, our within-participant comparison approach accounted for global differences arising from the DTI protocol and gradient scheme, as well as other global effects arising from developmental differences. Fourthly, DTI images were acquired using images from a single scanner vendor. However, previous studies have shown that the FA values do not vary significantly between different vendors [48], thus supporting the broad applicability of our results. Fifthly, only one-half of our patients had histologically proven FCD. Nevertheless, the strict radiological inclusion and exclusion criteria used in our study allowed the selection of a patient cohort with lesions highly suggestive of an FCD. Finally, the absence of MRI-negative patients in our study limits the possibility to extend our results to this patient cohort. However, we are planning to validate our findings in a larger cohort of patients including MRI-negative cases.

## Conclusion

Our study observed significant differences in FA and MD between FCD and contralateral brain parenchyma, suggesting that DTI may prove to be a useful tool for FCD characterisation by imaging FCD-associated microstructural changes. In particular, FA proved to be the most sensitive metric for differentiating FCD from CBP and distinguishing between FCD types. A positive history of SE increased the magnitude of FA difference values, likely reflecting the histological changes induced in the brain by such a destructive event. Future studies are needed to further explore the potential of DTI for presurgical FCD profiling, integrating this new biomarker in epilepsy imaging protocols and automatic lesion detection tools, ultimately aiming to improve seizure and cognitive outcomes in pharmacoresistant paediatric focal epilepsy.

## Supplementary Information

**Additional file 1.** MRI post-processing, supplementary tables, and figure.

## Data Availability

The dataset generated and/or analysed during the current study are not publicly available because it is based on 3D imaged which might easily showcase the identity of the patients but are available from the corresponding author on reasonable request.

## Abbreviations

ASD: Anti-seizure drugs
CBP: Contralateral brain parenchyma
FCD: Focal cortical dysplasia
SE: Status epilepticus
FA: Fractional anisotropy
MD: Mean diffusivity
AD: Axial diffusivity
RD: Radial diffusivity
SD: Standard deviation
IQR: Interquartile range
CI: Confidence interval
NODDI: Neurite orientation dispersion and density imaging
SMT: Spherical mean technique
MRI: Magnetic resonance imaging
DTI: Diffusion tensor imaging
ROI: Region of interest
T1WI: T1-weighted images
T2WI: T2-weighted images
FLAIR: Fluid-attenuated inversion recovery
FSPGR: Fast spoiled gradient echo

## References

[1] Moreau JF, Fink EL, Hartman ME (2013). Hospitalizations of children with neurologic disorders in the United States. Pediatr Crit Care Med.

[2] Wirrell EC (2013). Predicting pharmacoresistance in pediatric epilepsy. Epilepsia.

[3] Kwan P, Schachter SC, Brodie MJ (2011). Drug-resistant epilepsy. N Engl J Med.

[4] Ramantani G, Reuner G (2018). Cognitive development in pediatric epilepsy surgery. Neuropediatrics.

[5] Wirrell E, Wong-Kisiel L, Mandrekar J, Nickels K (2012). Predictors and course of medically intractable epilepsy in young children presenting before 36 months of age: a retrospective, population-based study. Epilepsia.

[6] Dwivedi R, Ramanujam B, Chandra PS (2017). Surgery for drug-resistant epilepsy in children. N Engl J Med.

[7] Blumcke I, Spreafico R, Haaker G (2017). Histopathological findings in brain tissue obtained during epilepsy surgery. N Engl J Med.

[8] Kadish NE, Bast T, Reuner G (2019). Epilepsy surgery in the first 3 years of life: predictors of seizure freedom and cognitive development. Clin Neurosurg.

[9] Ramantani G, Kadish NE, Strobl K (2013). Seizure and cognitive outcomes of epilepsy surgery in infancy and early childhood. Eur J Paediatr Neurol.

[10] Bast T, Ramantani G, Seitz A, Rating D (2006). Focal cortical dysplasia: prevalence, clinical presentation and epilepsy in children and adults. Acta Neurol Scand.

[11] Rowland NC, Englot DJ, Cage TA (2012). A meta-analysis of predictors of seizure freedom in the surgical management of focal cortical dysplasia. J Neurosurg.

[12] Guerrini R, Duchowny M, Jayakar P (2015). Diagnostic methods and treatment options for focal cortical dysplasia. Epilepsia.

[13] Kim YH, Kang H-C, Kim D-S (2011). Neuroimaging in identifying focal cortical dysplasia and prognostic factors in pediatric and adolescent epilepsy surgery. Epilepsia.

[14] Blümcke I, Thom M, Aronica E (2011). The clinicopathologic spectrum of focal cortical dysplasias: a consensus classification proposed by an ad hoc Task Force of the ILAE Diagnostic Methods Commission. Epilepsia.

[15] Eriksson SH, Rugg-Gunn FJ, Symms MR (2001). Diffusion tensor imaging in patients with epilepsy and malformations of cortical development. Brain J Neurol.

[16] Rau A, Kellner E, Foit NA (2019). Discrimination of epileptogenic lesions and perilesional white matter using diffusion tensor magnetic resonance imaging. Neuroradiol J.

[17] Hong S-J, Bernhardt BC, Caldairou B (2017). Multimodal MRI profiling of focal cortical dysplasia type II. Neurology.

[18] Lorio S, Adler S, Gunny R (2020). MRI profiling of focal cortical dysplasia using multi-compartment diffusion models. Epilepsia.

[19] Widjaja E, Zarei Mahmoodabadi S, Otsubo H (2009). Subcortical alterations in tissue microstructure adjacent to focal cortical dysplasia: detection at diffusion-tensor MR imaging by using magnetoencephalographic dipole cluster localization. Radiology.

[20] Diehl B, Tkach J, Piao Z (2010). Diffusion tensor imaging in patients with focal epilepsy due to cortical dysplasia in the temporo-occipital region: electro-clinico-pathological correlations. Epilepsy Res.

[21] Arain M, Haque M, Johal L (2013). Maturation of the adolescent brain. Neuropsychiatr Dis Treat.

[22] Toga AW, Thompson PM, Sowell ER (2006). Mapping brain maturation. Trends Neurosci.

[23] Bennett SH, Kirby AJ, Finnerty GT (2018). Rewiring the connectome: evidence and effects. Neurosci Biobehav Rev.

[24] Urbach H, Kellner E, Kremers N (2022). MRI of focal cortical dysplasia. Neuroradiology.

[25] Prada F, Gennari AG, Del Bene M (2019). Intraoperative ultrasonography (ioUS) characteristics of focal cortical dysplasia (FCD) type II b. Seizure.

[26] Lebel C, Treit S, Beaulieu C (2019). A review of diffusion MRI of typical white matter development from early childhood to young adulthood. NMR Biomed.

[27] Maiworm M, Nöth U, Hattingen E (2020). Improved visualization of focal cortical dysplasia with surface-based multiparametric quantitative MRI. Front Neurosci.

[28] de Fonseca V, C, Yasuda CL, Tedeschi GG,  (2012). White matter abnormalities in patients with focal cortical dysplasia revealed by diffusion tensor imaging analysis in a voxelwise approach. Front Neurol.

[29] Wang Y, Zhou Y, Wang H (2018). Voxel-based automated detection of focal cortical dysplasia lesions using diffusion tensor imaging and T2-weighted MRI data. Epilepsy Behav.

[30] Colciaghi F, Finardi A, Frasca A (2011). Status epilepticus-induced pathologic plasticity in a rat model of focal cortical dysplasia. Brain J Neurol.

[31] Amunts K, Zilles K (2015). Architectonic mapping of the human brain beyond Brodmann. Neuron.

[32] Fischl B, Dale AM (2000). Measuring the thickness of the human cerebral cortex from magnetic resonance images. Proc Natl Acad Sci U S A.

[33] Salo RA, Miettinen T, Laitinen T (2017). Diffusion tensor MRI shows progressive changes in the hippocampus and dentate gyrus after status epilepticus in rat—histological validation with Fourier-based analysis. Neuroimage.

[34] Sierra A, Laitinen T, Gröhn O, Pitkänen A (2015). Diffusion tensor imaging of hippocampal network plasticity. Brain Struct Funct.

[35] Ndode-Ekane XE, Hayward N, Gröhn O, Pitkänen A (2010). Vascular changes in epilepsy: functional consequences and association with network plasticity in pilocarpine-induced experimental epilepsy. Neuroscience.

[36] Budde MD, Janes L, Gold E (2011). The contribution of gliosis to diffusion tensor anisotropy and tractography following traumatic brain injury: validation in the rat using Fourier analysis of stained tissue sections. Brain J Neurol.

[37] Donkels C, Pfeifer D, Janz P (2017). Whole transcriptome screening reveals myelination deficits in dysplastic human temporal neocortex. Cereb Cortex.

[38] Lamberink HJ, Otte WM, Blümcke I (2020). Seizure outcome and use of antiepileptic drugs after epilepsy surgery according to histopathological diagnosis: a retrospective multicentre cohort study. Lancet Neurol.

[39] Blumcke I, Cendes F, Miyata H (2021). Toward a refined genotype-phenotype classification scheme for the international consensus classification of Focal Cortical Dysplasia. Brain Pathol.

[40] Blümcke I, Coras R, Busch RM (2021). Toward a better definition of focal cortical dysplasia: an iterative histopathological and genetic agreement trial. Epilepsia.

[41] Perry MS, Shandley S, Perelman M (2022). Surgical evaluation in children < 3 years of age with drug-resistant epilepsy: patient characteristics, diagnostic utilization, and potential for treatment delays. Epilepsia.

[42] Hildebrandt M, Pieper T, Winkler P (2005). Neuropathological spectrum of cortical dysplasia in children with severe focal epilepsies. Acta Neuropathol.

[43] Ramantani G, Kadish NE, Brandt A (2013). Seizure control and developmental trajectories after hemispherotomy for refractory epilepsy in childhood and adolescence. Epilepsia.

[44] Ramantani G, Strobl K, Stathi A (2013). Reoperation for refractory epilepsy in childhood: a second chance for selected patients. Neurosurgery.

[45] Ramantani G (2019). Epilepsy surgery in early life: the earlier, the better. World Neurosurg.

[46] Ni H, Kavcic V, Zhu T (2006). Effects of number of diffusion gradient directions on derived diffusion tensor imaging indices in human brain. AJNR Am J Neuroradiol.

[47] Jones DK (2004). The effect of gradient sampling schemes on measures derived from diffusion tensor MRI: a Monte Carlo study. Magn Reson Med.

[48] Min J, Park M, Choi JW (2018). Inter-vendor and inter-session reliability of diffusion tensor imaging: implications for multicenter clinical imaging studies. Korean J Radiol.

