# Supplementary material for: Diffusion tensor imaging discriminates focal cortical dysplasia from normal brain parenchyma and differentiates between focal cortical dysplasia types
Source: Insights Imaging. 2023 Feb 24;14:36. doi: 10.1186/s13244-023-01368-y (PMC9958211; doi:10.1186/s13244-023-01368-y)
Supplement: Supplementary file 1 — Additional file 1. MRI post-processing, supplementary tables, and figure. [file 13244_2023_1368_MOESM1_ESM.pdf]

ELECTRONIC SUPPLEMENTARY MATERIAL

Diffusion Tensor Imaging discriminates focal cortical dysplasia from normal brain parenchyma and differentiates between focal cortical dysplasia types

MRI post-processing

FSL (www. fmrib.ox.ac.uk/fsl) was used to pre-process both 3D FSPGR T1 and DTI images. Fslroi, swapdim, and bet functions were used to crop (excluding the patient's neck), reorient, and skull-strip the T1 image volume. Pre-processing of DTI images included eddy current correction, skull stripping, and diffusion tensor model fitting, leading to the generation of FA, MD, AD, and RD maps. Fslroi was used to remove b0 images from the eddy current corrected DTI data before averaging the non-b0 images into a mean b1000 image. The latter was co-registered to the skull-stripped T1-weighted image volume using a rigid body registration for the initial alignment of the images. Subsequently, a nonlinear registration was performed using reg_f3d (Niftyreg), allowing the mean b1000 images to be co-registered to the skull-stripped T1 weighted images. FA, MD, AD, and RD maps were aligned to the T1 images by applying the inverse transformation from the previously generated matrix. The FA, MD, AD, and RD values within each FCD were computed after overlaying the previously generated binary mask on each DTI map. The skull-stripped T1 weighted images were further rigidly aligned to the adult 1 mm MNI-skull-stripped atlas to enable flipping the lesion label symmetrically across the midline, while avoiding label or image deformation. The same transformation was also applied to the DTI maps and the binary masks (**Fig. 2**), the 3D binary mask was flipped across the midline, its position was thoroughly checked, and the values of the CBP extracted.

		Parameters								
		TE (msec)	TR (msec)	TI (msec)	Flip angle	Averages	Slice thickness (mm)	Spacing between slices (mm)	FOV (mm)	Voxel size (mm)
Sequences	3D FSPGR T1	5	12	500	12°	1	1	1	240 x 192	0.5 x 0.5
	2D T2 weighted	111	5732	-	111°	2	3	3.3	200 to 240 x 160 to 192	0.43 x 0.43
	2D FLAIR	120	9463	2329	111°	1	3	3.6	200 to 240 x 200 to 240	0.43 x 0.43
	3D FLAIR	130	7002	1905	90°	1	1.2	0.6	256 x 192	0.5 x 0.5
	DTI 21 directions	101	10800	-	90°	1	3	3	240 x 240	0.94 x 0.94
	DTI 35 directions	92	5331	-	90°	1	3	3	220 x 220	0.94 x 0.94

Supplementary Table 1. Image parameters.

TE: Echo time; TR: Repetition time; TI: Inversion time; FOV: Field-of-view; msec: milliseconds; mm: millimeter; 3D: Three-dimensional; 2D: Two-dimensional; FSPGR: Fast spoiled gradient echo; FLAIR: Fluid attenuated inversion recovery; DTI: Diffusion tensor imaging

N.	Patient Characteristics									Lesion location		Surgical data	
	Sex	Age at epilepsy onset (y)	Age at MRI (y)	Seizure frequency at MRI (seizures/month)	Focal to bilateral tonic-clonic seizures or epileptic spasms	Family history of epilepsy	History of febrile seizures	History of status epilepticus	Additional diagnoses	Hemisphere involved	Lobe involved	Epilepsy surgery	FCD type*
1	M	0	0	4	Yes	No	No	Yes	-	Right	Frontal	Yes	IIa
2	F	0	10	3	No	No	No	No	West Syndr.	Right	Temporal	Yes	IIa
3	M	4	8	0	No	No	No	No	West Syndr.	Right	Frontal	No	-
4	F	3	6	1	Yes	No	No	No	-	Right	Frontal	Yes	IIb
5	M	2	3	300	No	No	No	No	-	Left	Frontal	Yes	I
6	M	1	2	1	No	No	No	No	-	Right	Parietal	No	-
7	M	0	13	0	No	No	No	No	-	Right	Frontal	No	-
8	M	0	2	0,3	No	No	Yes	No	-	Right	Frontal	No	-
9	M	2	2	360	No	No	No	No	-	Right	Temporal	Yes	I
10	M	0	2	16	No	No	No	No	West Syndr.	Left	Frontal	Yes	I
11	F	13	15	12	Yes	Yes	No	No	-	Right	Temporal	Yes	IIIb
12	M	5	12	2	Yes	No	No	No	-	Left	Parietal	Yes	IIb
13	F	0	2	6	No	No	No	No	Genetic var.	Right	Multilobar	Yes	I
14	M	0	1	150	Yes	No	No	No	Genetic var.	Left	Frontal	Yes	I
15	M	0	3	300	No	Yes	Yes	No	-	Left	Temporal	Yes	I
16	F	0	2	16	No	No	Yes	No	-	Right	Frontal	Yes	IIa
17	F	2	3	60	No	No	No	No	-	Left	Frontal	Yes	I
18	M	4	8	160	Yes	No	Yes	No	-	Left	Temporal	No	-
19	F	9	11	0	No	No	No	No	-	Right	Multilobar	No	-
20	F	1	1	300	No	No	No	No	-	Left	Frontal	No	-
21	M	8	8	900	No	No	No	Yes	-	Left	Multilobar	No	-
22	M	1	4	20	No	Yes	Yes	Yes	-	Right	Frontal	No	-
23	F	0	4	30	Yes	No	No	No	Genetic var., CSWS	Right	Frontal	Yes	IIa
24	M	0	2	900	Yes	Yes	No	Yes	-	Right	Frontal	Yes	IIb
25	M	4	4	900	No	No	No	Yes	Genetic var.	Right	Temporal	No	-
26	F	2	11	3	Yes	No	Yes	No	Genetic var.	Right	Temporal	No	-
27	F	2	3	0	Yes	Yes	Yes	Yes	-	Left	Parietal	No	-
28	F	1	3	0	No	Yes	No	No	-	Left	Parietal	No	-
29	M	11	13	12	No	No	No	No	-	Right	Multilobar	No	-
30	M	0	16	0	Yes	No	No	No	West Syndr.	Right	Frontal	No	-
31	M	0	11	0	Yes	Yes	No	Yes	-	Left	Occipital	No	-
32	M	8	8	0	No	Yes	No	No	CSWS	Left	Frontal	No	-

Supplementary Table 2. Detailed patient demographics.

M: Male; F: Female; y: years; MRI: Magnetic resonance imaging; FCD: Focal cortical dysplasia; CSWS: Continuous spike and wave during sleep; West syndr.: West syndome; Genetic var.: pathogenic genetic variant; *: according to histopathology in surgical patients.

Lesion characteristics			Non-surgical patients (N = 17)	Surgical patients (N = 15)	*p*-value	All patients (N = 32)
Lateralization, n (%)					1.0	
	Right		10 (59%)	9 (60%)		19
	Left		7 (41%)	6 (40%)		13
Localisation, n (%)					0.66	
	Temporal		3 (18%)	4 (26%)		7 (22%)
	Frontal		7 (41%)	9 (60%)		16 (50%)
	Posterior		4 (23%)	1 (7%)		5 (16%)
		Parietal	3 (75%)	1 (100%)		4 (13%)
		Occipital	1 (25%)	0 (0%)		1 (3%)
	Multilobar§		3 (18%)	1 (7%)		4 (12%)
Volume in mm^3, median (IQR)					0.54	
	Temporal		1272 (177 - 17934)	16910 (4684 - 31296)		11309 (1272 - 22510)
	Frontal		3935 (3404 - 9427)	7591(5668 - 43921)		7144 (4146 - 11741)
	Posterior		5556 (4115 - 19911)	5986 (/)		5986 (4558 - 13073)
		Parietal	5001 (4115 - 6234)	5986 (/)		5494 (4337 - 6172)
		Occipital	19911 (/)	/		19911 (/)
	Multilobar§		5556 (2962 - 25549)	43265 (/)		15553 (3616 - 38836)

Supplementary Table 3. Lesion characteristics of our cohort.

N: number; IQR: interquartile range; §the precise lesion localisation was: one fronto-temporo-insular, two occipito-temporal, and one fronto-parietal.

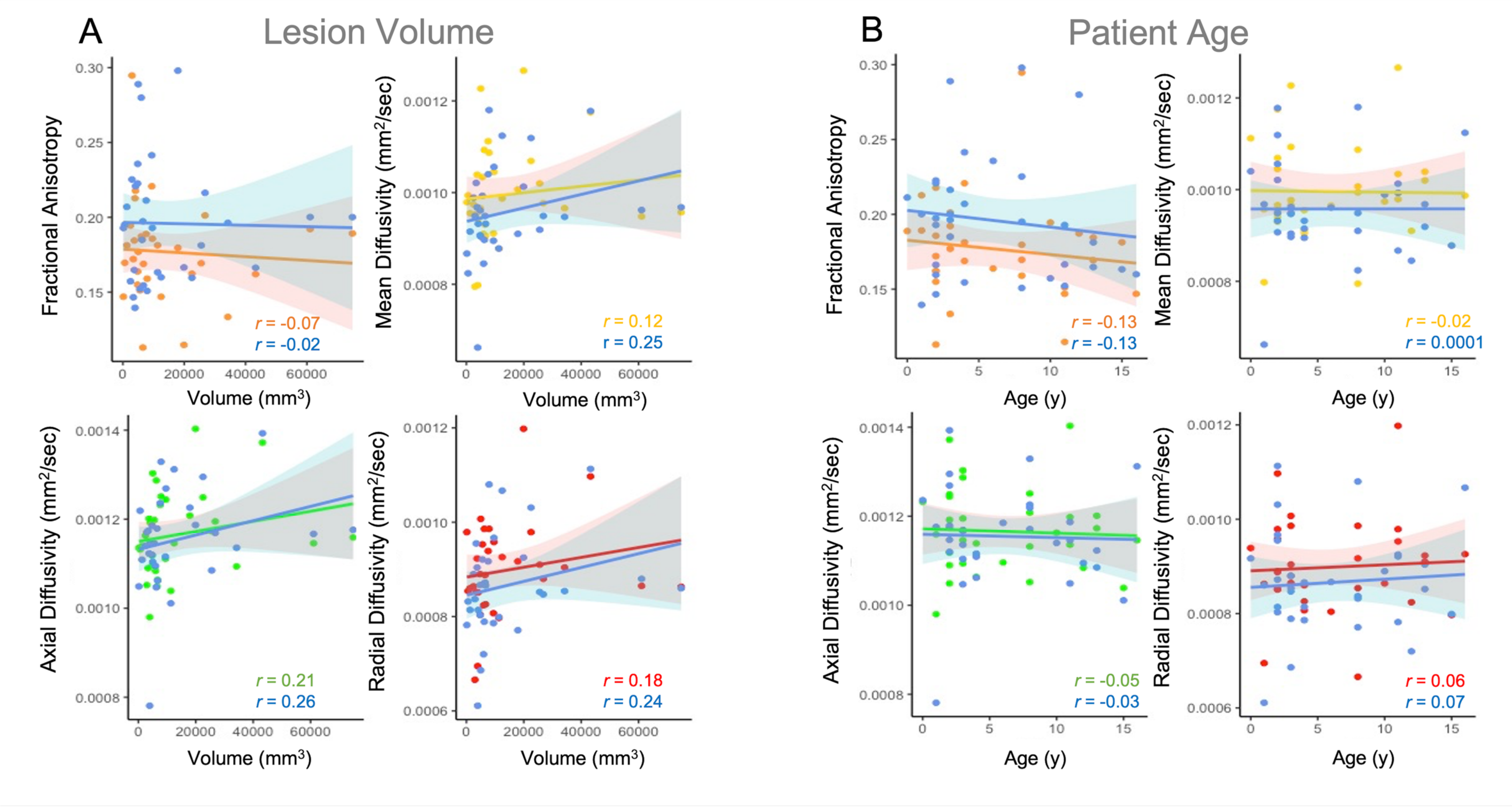

Supplementary Figure 1: **Scatter plots illustrating the correlation between DTI scalar indices and (A) lesion volume, as well as (B) patient age.**

None of the DTI scalar indices, calculated in FCD or in CBP, showed a significant correlation with lesion volume (A) or patient age (B). The colors of the dots and regression lines match those in Figure 3, with orange (FA), yellow (MD), green (AD), and red (RD) representing FCD values, and blue representing CBP values.

FCD: Focal cortical dysplasia; CBP: Contralateral brain parenchyma; FA: Fractional anisotropy; MD: Mean diffusivity; AD: Axial diffusivity; RD: Radial diffusivity; *r*: Pearson's coefficient.
